# A Micromechanical Wide-Range Stiffness-Tuning Mechanism for MEMS Optical Switches

**DOI:** 10.3390/mi16040397

**Published:** 2025-03-28

**Authors:** Tongtian Zhang, Junhui Wu, Guangya Zhou

**Affiliations:** Department of Mechanical Engineering, National University of Singapore, Singapore 117575, Singapore

**Keywords:** stiffness adjustment, MEMS, tunable stiffness, compliant mechanism

## Abstract

MEMS stiffness-tunable devices, owing to their low resonant frequency and high sensitivity, have been widely adopted in fields such as biological force sensing, vibration sensing, and inertial sensing. However, traditional stress-effect-based stiffness-adjustment methods offer limited tuning range. This paper introduces a novel stiffness-tuning mechanism based on the principle of stiffness compensation, integrating positive stiffness springs with V-shaped negative stiffness springs in a parallel configuration. A self-locking mechanism enables precise control of the mechanical preloading on the negative stiffness structures to realize stiffness adjustment. This design is prototyped by microscale fabrication techniques and is suitable for miniaturization. The experimental results confirm a stiffness reduction of over 90% and demonstrate bistability. These findings highlight the potential of the design for high-sensitivity MEMS accelerometers and dual-mode optical switches with low switching voltage.

## 1. Introduction

Tunable stiffness mechanisms (TSM) exhibit significant advantages, including high energy efficiency, multimodal adaptability, and low stiffness. These features make TSM highly versatile, with applications such as space machinery [1], soft robotics [2], vibration isolators [3,4,5], and impact absorption systems [6]. In the field of micro-electromechanical systems (MEMS), TSM are also promising in devices such as optical switches [7], MEMS graspers [8], high-Q mechanical resonators [9,10], and high sensitivity accelerometers [11,12]. Stiffness tuning in MEMS devices is primarily realized by three approaches [13]: electrostatic force modulation [14,15], structural modification of plate springs, and stress effect-based deformation. While the first two methods provide significant stiffness variation, they suffer from high operating voltages and continuous powering. Meanwhile, the stiffness-tuning range of the stress-based approach is limited.

Recent studies have improved the stress-based method by incorporating stiffness compensation mechanisms, which introduce negative stiffness to counteract the inherent positive stiffness. This negative stiffness is generated through deformation-induced stress. For example, Kuppens et al. [16] and Liang et al. [17] experimentally validated negative stiffness by preloading plate beams of additively manufactured prototypes. However, maintaining external preloading presents challenges in MEMS applications.

In this paper, we report a novel micromechanical wide-range stiffness-tuning mechanism, incorporating a self-locking structure to control preloading. This design is compatible with micro- and nanoscale fabrication techniques, making it suitable for MEMS applications. A simplified model is established to verify the feasibility of the proposed principle, and the structure is designed and numerically studied using finite element analysis (FEA). Furthermore, a prototype is fabricated using silicon-on-insulator multi-user MEMS processes (SOIMUMPs). Experimental results of the dynamic performance tests validate the stiffness tunability. The bistability of the structure is then confirmed by a static performance test. This work provides a viable solution for high-sensitivity inertial sensors and MEMS optical switches with dual-mode operation requirements.

## 2. Design and Simulation

Stiffness compensation is achieved by combining negative stiffness and positive stiffness, which can be concluded by the two following equations:

(1) $$k_{\mathrm{total}}\left(x\right)=k_{-}\left(x\right)+k_{+}\left(x\right)$$

(2) $$F_{\mathrm{total}}\left(x\right)=F_{-}\left(x\right)+F_{+}\left(x\right)$$

**Figure 1:**
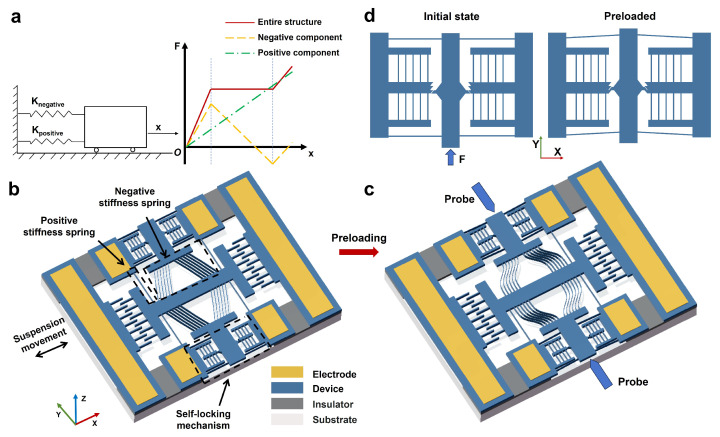
(**a**) Illustration of force (*F*) versus displacement (*x*) behaviors of the tunable stiffness mechanism (solid line), a positive stiffness mechanism (dashed–dotted line), and negative stiffness component (dashed line); (**b**) schematic of the TSM; (**c**) schematic of the TSM after preloading via a probe; (**d**) two different states of the self-locking mechanism.

Equation (2) is derived by integrating Equation (1), where *x* represents the displacement from the initial state. The force–displacement curve based on Equation (2) is shown in Figure 1a. A cart connected to both negative and positive stiffness components moves along the x-direction from its initial position. As illustrated, the regions of positive and negative stiffness overlap such that the net force is nearly zero within a certain range. To generate the required negative stiffness, a preload method is implemented, as described in Section 2.1. Figure 1b illustrates the schematic of the proposed mechanism, comprising four main components: the positive stiffness unit, the negative stiffness unit, a self-locking mechanism for preloading control, and comb drive actuators. Additionally, Figure 1c presents the TSM after preloading with a probe.

### 2.1. Stiffness-Adjustment Method

**Figure 2:**
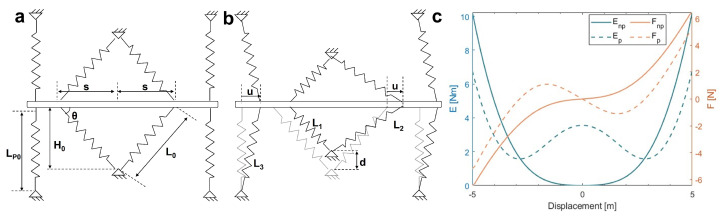
(**a**) Initial state of the model; (**b**) preloaded state; (**c**) relationship between elastic potential energy (*E*) and force (*F*) with (dashed line) and without (solid line) preloading.

To explain the stiffness-tuning method, a simplified model consisting of springs and a movable rod is constructed, as shown in Figure 2. In Figure 2a, four vertically oriented springs represent the positive stiffness component, while two sets of V-shaped springs represent the negative stiffness component without deformation. These springs are fixed at one end and attached to a movable rod at the other. Figure 2b illustrates the scenario where the V-shaped springs undergo vertical displacement *d*, indicating the application of preloading. In this state, the movable rod has a horizontal displacement *u*.

Let $L_{p0}$ and $L_{0}$ denote the original lengths of the positive and negative stiffness springs, respectively. Following a horizontal displacement *u*, the left and right negative stiffness springs extend to lengths $L_{1}$ and $L_{2}$, while the positive stiffness spring reaches a length of $L_{3}$. The distance between the V-shaped negative stiffness springs and the center is denoted as *s*. Let $H_{0}$ represent the distance between the initial fixing point of the negative stiffness spring and the rod, and the angle between the negative stiffness spring and the rod is θ. The geometric relationships are established as follows: $H_{1}=H_{0}-d,$$L_{0}=\sqrt{H_{0}^{2}+s^{2}},L_{1}=\sqrt{{\left(s-u\right)}^{2}+H_{1}^{2}},L_{2}=\sqrt{{\left(s+u\right)}^{2}+H_{1}^{2}},L_{3}=\sqrt{u^{2}+L_{p0}^{2}}$. According to Hooke’s law, the relationship between the system’s elastic potential energy *E* and the displacement *u* is expressed as follows:

(3) $$E\left(u\right)=k[{\left(L_{1}-L_{0}\right)}^{2}+{\left(L_{2}-L_{0}\right)}^{2}+2{\left(L_{3}-L_{P0}\right)}^{2}]$$

(4) $$F\left(u\right)=\frac{dE(u)}{du};$$

To perform calculations in MATLAB® using the provided equations, we take values: $H_{0}=4$ m, $L_{P0}=5$ m, s=1 m, d=2 m, k=0.5 N/m, −5≤u≤5 m.

The blue and yellow curves in Figure 2c represent the relationship between displacement and elastic potential energy and force, respectively. The dashed and solid lines correspond to conditions with and without preloading. The results show that preloading induces significant stiffness change over a certain displacement range. This confirms the feasibility of achieving stiffness variation through stiffness compensation. The energy curve also reveals two distinct minima in potential energy, which is indicative of bistability [18,19].

### 2.2. Simulation

Considering the mechanics of plate beams, the proposed model is extended and verified using FEA. The material properties used in the simulations are listed in Table 1. To reduce computational cost while ensuring accuracy, the simulations are performed in a two-dimensional physical field using free triangular mesh discretization. The total number of elements is 40,855, with an average element quality of 0.7997. A localized mesh refinement is applied to the slender beam regions, with a minimum element size of 0.044 μm. Furthermore, the simulations utilize the geometric nonlinear solver provided by the simulation software.

A parametric sweep is adopted to iteratively update critical geometric parameters. During this process, two key metrics are considered: the force–displacement relationship of the structure and the maximum principal stress. Based on empirical insights, the maximum stress is targeted to remain below 1 GPa to ensure that the plate springs would not fracture during deformation [20]. Each V-shaped beam is formed by two plate beams connected in series with a high-curvature transition section between them. This configuration ensures that the beams bend outward as intended.

To further reduce the stress, six identical V-shaped beams are connected in parallel on each side. Compared with a single set of V-shaped beams, this configuration requires less preloading to achieve sufficient negative stiffness that counteracts positive stiffness, thereby preventing fracture during large deformations of the slender beams. The design parameters are as follows: the thickness of the device layer is 25 μm, and the width of all plate beams is 4 μm. Each V-shaped beam consists of two plate beams, each 400 μm in length, and connected by a curvature radius of 1200 μm. The spacing between adjacent V-shaped beams is 30 μm, and the angle between the beams and the central truss is 67.5°. The length of the four straight beams providing positive stiffness is 1000 μm.

**Figure 3:**
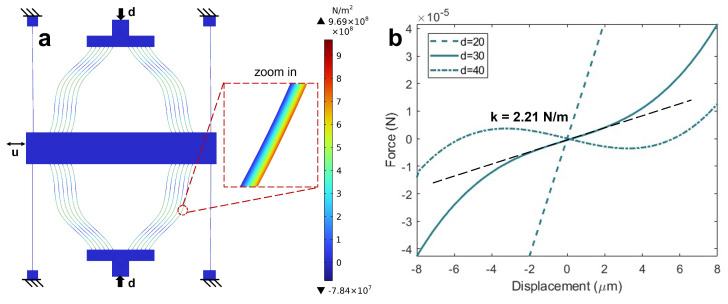
Simulation results: (**a**) stress distribution map obtained from FEA and the associated boundary condition; (**b**) force–displacement relationship curves under varying preload displacements (*d*) of 20 μm (dashed line), 30 μm (solid line), and 40 μm (dashed–dotted line).

The simulation results are shown in Figure 3. As illustrated in Figure 3a, the maximum stress is less than 1 GPa. Figure 3b illustrates the variation in stiffness at different loading distances *d*. When the loading distance reaches 30 μm, the stiffness is approximately 2.21 N/m. Additionally, the device exhibits bistable behavior at d=40 μm.

### 2.3. Other Design Considerations

**Figure 4:**
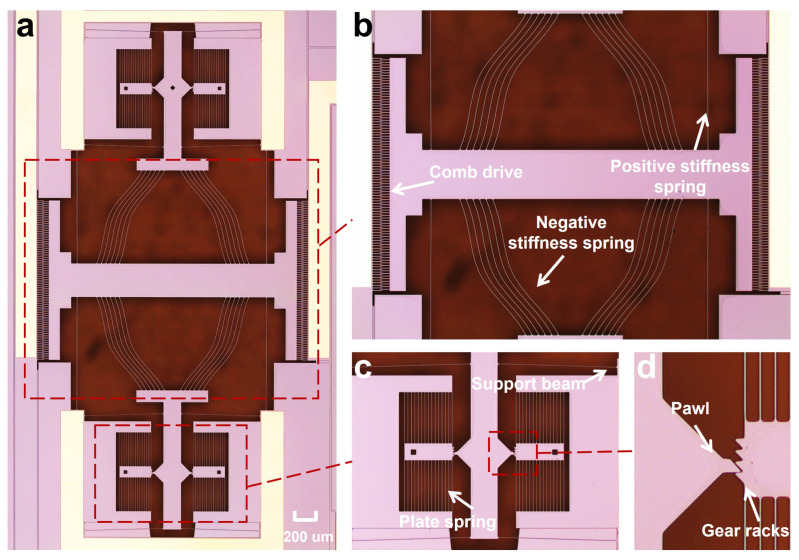
Microscope images captured under the preloading condition of Step 1, showing the (**a**) overall device, (**b**) positive and negative stiffness spring, (**c**) self-locking mechanism, and (**d**) ratchet gear racks and pawl.

The preloading of the device is achieved through a self-locking mechanism, as illustrated in Figure 1d and Figure 4c,d. This mechanism operates similarly to a ratchet system and is composed of two ratchet gear racks and a pawl. Each ratchet gear rack contains four teeth and is designed to exhibit low stiffness in the transverse direction while maintaining high stiffness longitudinally to allow controlled engagement. The pawl is integrated into a central guide beam, which is supported by four auxiliary beams to prevent rotational motion. When a probe applies force to the guide beam, the pawl moves forward and securely engages with the gear racks, locking its position to prevent backward motion.

A comb-drive actuator is integrated to generate the electrostatic force for subsequent experimental testing. To avoid the pull-in phenomena, the comb-drive design is based on a validated approach [21], and substantiated by simulation results. The detailed parameters of the two structures are summarized in Table 2.

## 3. Prototype and Experiment

The prototype is fabricated using SOIMUMPs, with detailed fabrication steps available in the handbook [22]. Microscope images of the fabricated structure are shown in Figure 4. A dimensional adjustment was made to the self-locking mechanism to ensure proper functionality. As mentioned in Table 2, the standard spacing between the teeth of the rack gear is 15 μm. However, during fabrication, if the initial gap between the pawl and the first tooth is too small, resolution limitations may cause them to adhere, hindering the preloading step. To mitigate this issue, the initial step distance is increased to 18.89 μm.

**Figure 5:**
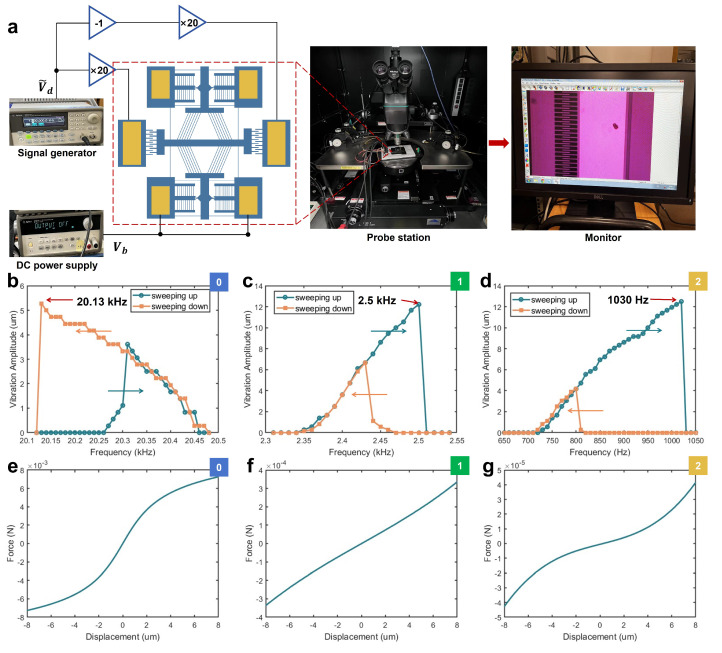
(**a**) Experimental setup; (**b**–**d**) frequency response curves from upward and downward sweeps at varying preload conditions (Steps 0–2, as indicated in the upper right corner of the figure); each display distinct behaviors: softening, hardening, and hardening effect; (**e**–**g**) force–displacement curves obtained from FEA under identical preload conditions were used to fit the experimental (**b**–**d**) data reflecting nonlinear behavior.

We construct the driving circuit shown in Figure 5a to measure the stiffness of our device. Equation (5) presents the equation of motion (EOM) for the MEMS comb-drive resonator. Based on the resonant frequency of the MEMS comb-drive resonator, the stiffness is calculated by Equation (6).

(5) $$m\ddot{x}+c\dot{x}+kx=4\frac{n\epsilon t}{g}V_{b}V_{d}\cos\left(\omega_{d}t\right)$$

(6) $$k=\omega^{2}m_{eff}$$

where $\omega_{d}$ is the angular frequency of the alternating current, ω is the natural angular frequency of the device, and $m_{eff}$ denotes the effective mass of the suspensions. During the experiment, the DC bias voltage $V_{b}$ is applied at one side of ratchet while the signal generator outputs a sine signal with modulated frequency and amplitude. The frequency is swept both upward and downward, each with a step size of 10 Hz. Images of the device are captured using an optical microscope and CCD camera. When the device vibrates, its motion produces ghost images that is visible on the monitor. The vibration amplitude is measured with these images. The experiments are conducted under 0, 18.89 μm, and 33.89 μm preload conditions, with frequency swept sequentially. Based on the displacement increments caused by the self-locking mechanism, the steps are referred to as Step 0, Step 1, and Step 2, respectively. Under the Step 0 condition, DC voltage $V_{b}=40$ V and the AC voltage amplitude $V_{d}=1$ V. Under Step 1, $V_{b}=10$ V and $V_{d}=0.15$ V. Under Step 2, $V_{b}=10$ V and $V_{d}=0.03$ V. The experimental results are presented in Figure 5b–d.

The experimental results reveal nonlinear behavior, which has been widely studied in MEMS comb-drive resonators. According to research [23], the observed response without preload is characteristic of a spring softening effect, while preload in Steps 1 and 2 provide a hardening effect, which is consistent with the simulation results shown in Figure 5e–g. The device frequency decreases from 20.13 kHz to 1.03 kHz. The effective mass is calculated to be $3.767\times 10^{-8}$ kg. The stiffness is decreased from 602.62 N/m to 1.58 N/m, a reduction of 99.7%.

**Figure 6:**
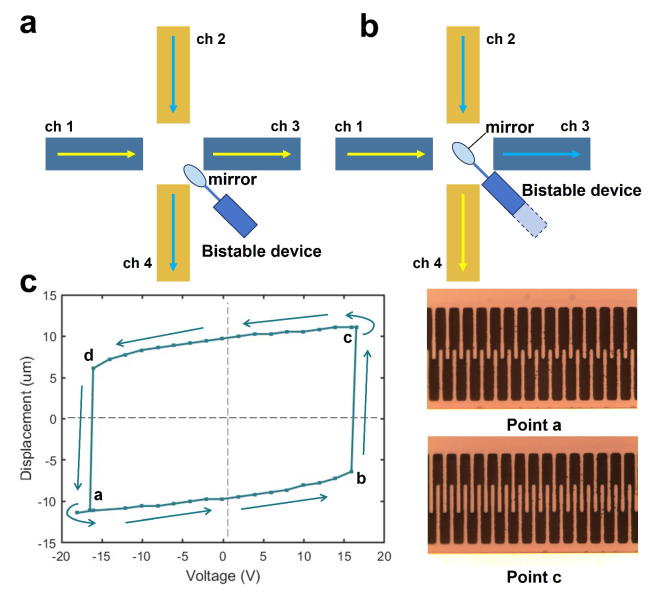
(**a**) Optical switch state 1; (**b**) optical switch state 2; (**c**) bistability test result and two stable states of the comb drive.

To investigate the bistable behavior, we further enlarge the preloading displacements in Step 3 to increase the negative stiffness of the structure. In this step, the pawl has advanced 48.89 μm. The results indicate potential applications in 2 × 2 optical switches. As illustrated in Figure 6a, by integrating a bistable structure with a micromirror, positional changes can alter the signal output. The experimental results of the bistable behavior are shown in Figure 6c. The experimental setup is identical to the circuit shown in Figure 5a, with the signal generator replaced by a DC drive voltage. By adjusting the drive voltage, a reversible transition between two stable states is demonstrated.

## 4. Discussion and Conclusions

In Table 3, we compare our work with other stiffness-tunable mechanisms. The first study, also based on stiffness compensation, achieved significant stiffness variation. However, its prototype was 3D-printed using fused deposition modeling, and its asymmetric structure may shift under preloading when scaled to the microscale. The second study introduced a MEMS-based design that uses a thermal actuator for preloading, achieving control solely through electrical circuits, but demonstrated limited performance. The third approach utilized electrostatic capacitive tuning, which effectively minimizes stiffness but requires continuous charging. Our method achieves a higher stiffness variation ratio, but it relies on an external probe for preloading, posing challenges for high-level integration. Additionally, as with other externally loaded preloading methods, it is more sensitive to manufacturing tolerances, a limitation reported in several studies [11,24].

In conclusion, this paper presents a novel tunable stiffness MEMS structure, designed based on the principle of stiffness compensation. This approach employs preloading to introduce a negative stiffness component that counteracts the positive stiffness component. To validate the feasibility of this method on the microscale, we first develop an analytical model and conduct numerical simulations to study the tunability of the stiffness. The prototype is fabricated by SOIMUMPs, and its dynamic performance is tested using typical comb-drive actuators configured with a push–pull circuit. Experimental results demonstrate a significant reduction in the natural frequency of the device from 20.13 kHz to 1.03 kHz, corresponding to a 99.7% decrease in stiffness.

This innovative design features bistability and low-voltage actuation, making it well-suited for actuating micromirrors in optical switches. By controlling this structure, the signal transmission mode can be adjusted accordingly. Additionally, it also has potential applications in MEMS grippers requiring dual-mode functionality and high-sensitivity MEMS accelerometers.

## Figures and Tables

**Table 1:** Material data.

Density	Poisson’s Ratio	Young’s Modulus
2329 ${\mathrm{kg}/m}^{3}$	0.28	169 GPa

**Table 2:** Remaining geometrical parameters.

Part	Description	Value
Self-locking	Length of plate springs	300 μm
	Width of plate springs	3 μm
	Tooth spacing of the rack gears	15 μm
	Overlap of two teeth	8 μm
	Length of support beams	700 μm
	Width of support beams	4 μm
Comb-drive	Length of fingers	60 μm
	Width of fingers	5 μm
	Number of fingers	88
	Gap of two fingers	3 μm
	Overlap of two fingers	20 μm

**Table 3:** Performance comparison of similar studies.

Ref.	Scale	Method	Variation Range	Decrease Ratio
[16]	mm	Stiffness compensation	6700 N/m to 80 N/m	98.8%
[25]	μm	Thermoelectric actuator	NA	2.12%
[15]	μm	Comb-finger capacitor with a curved contour	2.64 N/m to 0.528 N/m	80%
This work	μm	Stiffness compensation	606.62 N/m to 1.58 N/m	99.7%

## Data Availability

Data are available from the corresponding authors G.Z. upon reasonable request.
